# Patient Experience and Surgical Outcomes of Botulinum Toxin A Treatment in Complex Abdominal Wall Hernias: A Retrospective Analysis

**DOI:** 10.3389/jaws.2026.15899

**Published:** 2026-02-17

**Authors:** Angelina Klein, Aliona Wöhler, Robert Schwab, Christoph Güsgen, Arnulf Willms, Sebastian Schaaf

**Affiliations:** Department of General, Visceral and Thoracic Surgery, German Armed Forces Central Hospital of Koblenz, Koblenz, Germany

**Keywords:** abdominal wall hernia, abdominal wall reconstruction, botulinum toxin A infiltration, patient reported outcome measures, preoperative conditioning

## Abstract

**Background:**

Botulinum toxin A (BTA) is increasingly used for preoperative conditioning in patients with large or complex abdominal wall hernias. Injection into the lateral abdominal muscles 4–6 weeks before surgery induces temporary muscular relaxation and facilitates primary fascial closure, even in extensive defects (EHS W3), potentially reducing the need for component separation. While surgical outcomes are well documented, data on patient-reported experiences during the preoperative period remain limited. This retrospective study evaluated patient-reported symptoms between BTA injection and surgery and analyzed surgical results in this cohort.

**Methods:**

Between 2018 and 2024, 50 patients with complex abdominal wall hernias received preoperative BTA treatment followed by surgical repair. Demographic and surgical data, as well as BTA-related complications, were analyzed descriptively. A retrospective questionnaire assessed subjective experiences from injection to surgery, focusing on pain, physical changes (e.g., abdominal contour, trunk stability), and functional impairments (e.g., breathing, urination, defecation).

**Results:**

The study included 31 men and 19 women (mean age 63.5 years, BMI 28 kg/m^2^). The mean transverse defect width was 12.06 cm, with an average area of 170.24 cm^2^. Thirty eight patients had W3 hernias according to EHS (≥10 cm), while BTA was also used in selected cases with smaller defects with complicating factors. No major BTA-related complications occurred; minor hematomas were observed. The mean interval between injection and surgery was 39 days. Primary fascial closure was achieved in all patients. Mesh reinforcement was used in all cases, most commonly in sublay position (n = 47). A transversus abdominis release was performed in 28 cases (52%), and anterior component separation in five. Twenty-two patients (44%) completed the questionnaire. Injection pain ranged from NRS 1–8, typically resolving within 1–3 days; three patients reported no pain. Eight noticed abdominal contour changes, and two reported altered trunk function. One patient experienced mild shortness of breath and another constipation; no urinary issues occurred.

**Conclusion:**

Preoperative BTA conditioning is a safe and effective adjunct for abdominal wall reconstruction in complex hernias. The treatment facilitates fascial closure, avoids major complications, and causes only minor, short-lived discomfort or functional limitations, maintaining overall quality of life in the preoperative phase.

## Introduction

Incisional hernias are common complications after abdominal surgery, with reported incidences of 10%–23% depending on follow-up duration [1–6]. Surgical repair of complex abdominal wall hernias (W3, ≥10 cm) remains challenging despite their recent definition by the European Hernia Society in 2024, as these procedures are technically demanding and associated with high morbidity and mortality, particularly when (anterior) component separation is required [6–9]. The primary aim of abdominal wall reconstruction is anatomical restoration with improvement of patients’ quality of life. To facilitate tension-free closure in complex cases, preoperative strategies such as progressive pneumoperitoneum and chemical component separation using Botulinum toxin A (BTA) have gained increasing attention [10]. First described in 2009, BTA induces a temporary, reversible paralysis of the lateral abdominal wall muscles, allowing medial fascial advancement and potentially avoiding more invasive component separation techniques associated with higher complication rates [11–13].

The use of BTA for large incisional hernias is currently limited to individual therapeutic attempts (off-label use), as no standardized injection protocol has yet been established [14, 15]. Additionally, although no specific international protocol has been accepted, some consensus proposals have been reported [16].

Moreover, there are no systematic data on how patients experience BTA therapy or whether physical or functional impairments occur between injection and surgery.

With the increasing emphasis on patient-centered endpoints in abdominal wall reconstruction, patient-reported outcome data are particularly relevant in the context of preoperative BTA, as its effects persist throughout the interval between injection and surgery. Recently, the first international survey has provided initial data on treatment tolerance and symptom burden following BTA [17].

In this context, this study contributes to this growing field of research by not only evaluating the patient experience, including physical changes, but also examining the surgical outcomes in our patient cohort who underwent preoperative abdominal wall conditioning with BTA for large hernias.

## Materials and Methods

Between 2018 and 2024, a total of 50 patients with complex abdominal wall hernias underwent preoperative conditioning of the lateral abdominal muscles using BTA. Patients with abdominal wall defects ≥8 cm in width were routinely treated with BTA as part of an individualized off-label treatment approach. Smaller defects complicated by additional risk factors according to the EHS-supported Delphi consensus were also treated [7]. The indication was based on the surgical expertise of the designated surgeons at the Hernia Surgery Reference Center and were carried out according to a standardized modified BTA protocol based on Zendejas et al. [18].

Injections were administered in an outpatient setting under sterile conditions approximately 4–6 weeks prior to the planned hernia repair, each patient received five ultrasound-guided injections per side into the lateral abdominal wall (500 IU Dysport®, Ipsen, Boulogne-Billancourt, France, diluted in 80 mL NaCl plus 20 mL Ropivacaine 0.75%). Three injections were placed along the anterior axillary line and two along the mid-axillary line. Targeted muscle layers included the *obliquus externus*, *obliquus internus*, and *transversus abdominis*, each infiltrated with 3.3 mL of the solution per muscle per injection site.

Exclusion criteria included age under 18 years, pregnancy or breastfeeding, metastatic malignancies in a palliative setting, local or systemic infections, and known hypersensitivity to BTA. Neurological conditions such as myasthenia gravis, Lambert-Eaton syndrome, amyotrophic lateral sclerosis, or peripheral neuropathies were also considered contraindications for BTA therapy.

The data collection included the following parameters: demographic and biometric data, comorbidities, the size of the fascial defect, BTA-related complications, surgical procedures performed, and component separation techniques used.

Postoperatively, patients were retrospectively interviewed during the hospital stay using a clinically based questionnaire with ten items (see Supplementary Appendix 1 “Patient Questionnaire–Botulinum Toxin A Injection” [17]). The questionnaire assessed patients’ experiences during BTA infiltration and during the four to 6 weeks preceding surgery. The questionnaire has not been validated.

Data were analyzed descriptively. Nominal data were presented using absolute numbers with percentages, and metric data were recorded using the mean. All analyses were performed using SPSS (version 25, IBM, Armonk, United States).

The Ethics Committee approved this retrospective study of the State of Rhineland-Palatinate (2021-16034). The studies were conducted in accordance with the local legislation and institutional requirements. Written informed consent for participation was not required from the participants or the participants’ legal guardians/next of kin in accordance with the national legislation and institutional requirements. Written informed consent was obtained from the individual(s) for the publication of any potentially identifiable images or data included in this article. Also, the study was registered in the German Clinic Trials Registry (DRKS00028557) according to the ICMJE standards.

## Results

### Patient Cohort and Preoperative Conditioning

During the study period, a total of 50 patients with large incisional hernias were conditioned with botulinum toxin A (BTA); no patients met any contraindications or rather exclusion criteria, so all could be included in the analysis. The cohort included 31 male and 19 female patients with a mean age of 63.5 years (25–83 years). The average body mass index (BMI) was 28 kg/m^2^ (18–38 kg/m^2^). All patients had undergone at least one and up to ten previous abdominal surgeries. The most common comorbidities included cardiovascular diseases such as arterial hypertension, coronary artery disease, atrial fibrillation, obesity, nicotine abuse, and diabetes mellitus. Table 1 show most common patients characteristics and comorbidities.

**TABLE 1:** Patient characteristics and comorbidities.

Comorbidities	%	n
Cardio-vascular diseases	68%	36
Diabetes mellitus	26%	14
COPD/OSAS	28%	15
Chronic renal disease	16%	8
Smoking	43%	23
History of malignant disease	19%	10
Adipositas		
BMI ≤29	57%	31
BMI 30-34,9	34%	18
BMI ≥35	9%	5
ASA <3	39%	21
ASA >3	61%	33

**FIGURE 1:**
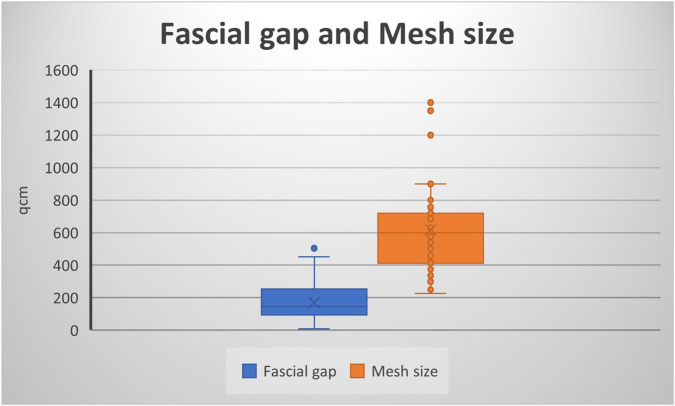
Fascial gap and Mesh size.

In 72% of the cases (n = 39), a midline hernia was present (including parts or the entire midline, e.g., M1-5, M2-4 etc.); four patients (7%) had lateral hernias, seven patients (13%) had midline and lateral hernia. The mean transverse hernia defect measured 12.06 cm (3–28 cm), the average vertical defect was 13.17 cm (3–28 cm), and the mean defect area was 170.24 cm^2^ (9–504 cm^2^, Figure 1). Five Patients had smaller defects <8 cm with complicating factors like a present stoma, off midline hernia, hernia recurrence with previous mesh implantation or parastomal plus midline hernia. In particular the patient with a 3 × 3cm defect (M5 W1) had a present urostomy (Mainz-Pouch) with umbilicus-stoma. A total of 38 patients had large transverse defects greater than or equal to 10 cm. Patients with W2 hernias (4–10 cm) routinely received BTA therapy when the defect measured approximately 8 cm or more. Hernia characteristics are shown in Table 2.

**TABLE 2:** Hernia characteristics according to EHS classification.

EHS-classification		n	% Of BTA-Patients
Midline	M1	2	3%
	M2	14	26%
	M3	32	59%
	M4	13	24%
	M5	6	11%
Lateral	L1	1	2%
	L2	2	3%
	L3	1	2%
Combined (midline + lateral)	M + L	7	13%
Width	W1	1	2%
	W2	11	20%
	W3	38	70%

### Operative Course and Surgery Associated Complications

The average interval between BTA injection and surgery was 39.24 days (28–65 days).

In the preoperative planning, an anatomical reconstruction with tension-free midline closure was planned in all cases. The surgical techniques employed included retromuscular sublay and open IPOM implantation, hybrid approaches such as video-assisted mini-open sublay (VAMOS), as well as anterior and posterior component separation. Primary fascial closure was achieved in 100% of cases. Overall, mesh implantation was performed in 50 patients. The mean mesh size was 617 cm^2^ (rage: 225–1,400 cm^2^, Figure 1).

Intraoperative fascial traction was required in five patients. A transversus abdominis release (TAR) was performed in 28 cases (52%), while anterior component separation was used in five patients; in 17 cases, primary fascial closure was achieved without additional techniques. Mesh reinforcement was predominantly performed in a sublay position (n = 47), using polyvinylidene fluoride (PVDF) meshes. A long-term absorbable monofilament mesh was used as an additional onlay mesh (n = 1) and in one case of lateral hernia.

Early postoperative complications occurred in 28% of patients (n = 15), mainly hematomas and seromas (Clavien–Dindo I–II). Clinically relevant complications (Clavien–Dindo IIIb) were observed in three patients, including one wound healing disorder and two abdominal wall infections, all managed successfully with vacuum therapy and mesh preservation [19].

### Botox-Associated Complications and Patient Feedback

No serious BTA-related complications (e.g., allergy, respiratory depression, bleeding, post-interventional bleeding, infection, sepsis) were observed. Only minor hematomas at the injection sites were reported. Twenty-two patients (44%) participated in the retrospective survey. The pain during the injection was rated between one and 8 Numerical rating scale (NRS) [20]. 86% (n = 19) reported pain lasting no more than 1–3 days, while three patients (n = 13%) experienced no pain at all. Eight patients (36%) noticed a change in abdominal shape, and two (9%) reported functional changes when sitting up or lying down. One patient each reported shortness of breath and difficulty with bowel movements. No issues with urination were reported. In response to the open-ended question regarding their experience with BTA injection, 13 patients (59%) reported positive memories and good tolerance of the treatment, 8 patients (36%) did not comment; one patient expressed surprise at the use of botulinum toxin in hernia surgery.

## Discussion

In our retrospective study, 50 patients with ventral incisional hernias who underwent preoperative treatment with botulinum toxin A (BTA) were analyzed over a seven-year period (2018-2024). Of these, 42% were obese with a body mass index (BMI) greater than 30 kg/m^2^. More than half of the patients had cardiovascular diseases and/or diabetes mellitus. All patients had at least one prior abdominal surgery; several had undergone multiple procedures.

These comorbidities resulted in morphologically complex or combined incisional hernias, often with large fascial defects, unstable abdominal wall structures, and altered anatomical conditions.

The size of our study population is comparable to other cohort studies [11, 18, 21–23].

According to the literature, obesity (BMI >30 kg/m^2^) is one of the most frequent comorbidities and risk factors in hernia surgery [24–26].

Surgical treatment of incisional hernias remains challenging. The primary goal is an anatomically correct and tension-free fascial closure, ideally supported by mesh reinforcement in the sublay position [27]. Transversus abdominis release (TAR) and/or anterior component separation (CS) is often required to achieve midline closure in large defects. Ibarra-Hurtado et al. reported performing anterior CS in 53% of their cases [22]. Bueno-Lledó et al. identified anterior CS and TAR as the most commonly used surgical approaches [25, 26, 28], while Nielsen et al. applied them in 40% of cases in 2020 [21]. Due to the morbidity associated with anterior CS—such as wound healing disorders, infections, or dehiscence [13] —and the technical limitations in achieving sufficient lateral mobilization, additional techniques may be necessary.

The use of BTA as a “chemical” or pharmacological component separation for large ventral hernias was first described by Ibarra-Hurtado et al. in 2009 and has since been widely adopted and modified [11]. The principle relies on temporary paralysis of the lateral abdominal muscles (external and internal obliques, and transversus abdominis) [12]. The ideal timing for administration remains undetermined; in the literature, BTA is usually applied 2–6 weeks before surgery [21, 23–26]. The maximum effect is believed to occur around 4 weeks after injection and gradually diminishes over the following months [13]. In our study, BTA was administered approximately 4–6 weeks prior to surgery.

Two pharmacological BTA preparations are commonly reported: Dysport® (Ipsen, France) and Botox® (Allergan, Ireland). A systematic review by Timmer et al. found Botox® to be used most frequently; five studies used Dysport®. Almost all authors describe ultrasound-guided injections into the external, internal oblique, and transversus abdominis muscles [13].

A high average fascial closure rate following preoperative BTA administration is documented in the literature [10, 29, 30]. Our findings support this, with a closure rate of 100%. Although this fascial closure rate cannot be attributed solely to the effect of BTA, it likely reflects the combined contribution of BTA and anterior and posterior component separation techniques. Importantly, BTA does result in fascial medialization, which may help reduce the need for anterior component separation, a procedure associated with increased morbidity [31]. This potential benefit may be explained, in part, by BTA-induced muscle relaxation and elongation of the lateral abdominal wall [22]. Based on four studies, Timmer et al. demonstrated a significant lateral elongation of the abdominal wall of up to 3.2 cm per side [13].

Rodriguez-Acevedo et al. reported mild BTA-related side effects in a survey of 27 patients, including occasional coughing, injection-site pain, superficial hematomas, and back pain [24]. Nielsen et al. observed injection pain in 2.7% of cases [21]. Larger cohorts from Bueno-Lledó et al. and Ibarra-Hurtado et al. also reported no BTA-related complications [22, 25]. This finding is further supported by a recent systematic review [13]. In our cohort, no serious adverse events occurred. Pain during (NRS 1-8) or shortly after injection was reported by patients and resolved within 1–3 days. In this context, the current protocol of five injections per side seems debatable. Two studies compared different BTA injection protocols: three sites in two muscles versus three sites in three muscles, and three sites versus two injection sites [32, 33]. No significant differences were observed between the groups, suggesting that two injections may be sufficient [32, 33]. However, there is still no evidence to support this, and further studies are required in the future.

There were no reports of functional limitations when sitting up or lying down. Bowel function was only minimally affected, and urination was not impaired. These results align with current literature.

However, cardiopulmonary complications like respiratory insufficiency and pneumonia were described by Zwaans et al. in 2024: The authors hypothesized that injection into the transversus abdominis muscle, which functions as an accessory respiratory muscle, may have contributed to the issue and recommend cardiopulmonary function tests [34]. While the impact of BTA on respiratory function has been a subject of discussion, prospective data based on spirometric assessment are now available and warrant consideration when evaluating the safety profile of this intervention in patients with large abdominal wall defects [17]. In our study, one patient—without a history of a chronic obstructive pulmonary disease or asthma—experienced mild respiratory impairment. As such, careful consideration should be given when administering BTA in patients with relevant pulmonary conditions, and in select cases, the transversus abdominis should be excluded from injection.

This study has several limitations. It is a single-center study without a control group, which limits the strength of the conclusions and precludes causal inference. The descriptive study design without comparative or statistical analyses further restricts the interpretability of the results and their generalizability. The sample size was small, and only 44% of patients completed the postoperative questionnaire. The reasons for the non-participation of the remaining patients are unknown, or they did not submit the questionnaire. Furthermore, no preoperative symptom assessment was performed prior to the BTA injection, which somewhat limits the evaluation of the extent of symptoms attributable to BTA. The questionnaire was completed postoperatively during the hospital stay, which may have led to memory and perception biases, possibly influenced by postoperative outcomes or complications. Finally, the heterogeneity of surgical techniques, despite high fascial closure rates, can be considered a further limitation. Despite these limitations, the study provides initial evidence that BTA can be safely used in this context. However, the results cannot be generalized, and further prospective, multicenter studies with larger samples and appropriate control groups are needed. Patient-reported outcomes in combination with surgical results should be further investigated.

### Conclusion

The study demonstrates that preoperative administration of Botulinum toxin A (BTA) into the abdominal wall appears to be a safe and effective method to improve outcomes in complex abdominal wall hernias and has minimal impact on patient-reported symptoms. Therefore, it would be desirable to establish clear guidelines for the use of Botox therapy in large abdominal wall hernias and to obtain official approval for this treatment in the future.

## Data Availability

The raw data supporting the conclusions of this article will be made available by the authors, without undue reservation.
