# Supplementary material for: Patient Experience and Surgical Outcomes of Botulinum Toxin A Treatment in Complex Abdominal Wall Hernias: A Retrospective Analysis
Source: J Abdom Wall Surg. 2026 Feb 17;5:15899. doi: 10.3389/jaws.2026.15899 (PMC12953170; doi:10.3389/jaws.2026.15899)
Supplement: Supplementary file 1 [file DataSheet1.docx]

**Patient Questionnaire – Botulinum Toxin A Injection** **[17]**

**1. Pain Assessment**

**1.1** How would you rate your pain during the injection of Botulinum toxin A into your abdomen?
*(0 = no pain, 10 = worst possible pain)*
**Scale (0–10)**

**1.2** How would you rate your pain the day after the injection? *(0 = no pain, 10 = worst possible pain)*
**Scale (0–10)**

**1.3** How was your pain during the week following the injection of Botulinum toxin A? *(0 = no pain, 10 = worst possible pain)*

**Scale (0–10)**

**1.4** If you experienced pain after the injection of Botulinum toxin A, how long did the pain last?

- 1–3 days
- 1 week
- 2 weeks
- 1 month
- 2 months
- Until the operation
- None

**2. Breathing**

**2.1** In the days and weeks following the injection of Botulinum toxin A and up until the day of your surgery, did you notice any changes in your breathing?

- No change.
- I was slightly short of breath but was still able to carry out my normal activities.
- I was noticeably more short of breath than usual and was only able to carry out my normal activities to a limited extent.
- I had severe shortness of breath and was therefore only able to carry out my normal activities to a limited extent.

**3. Abdominal Appearance**

**3.1** In the days and weeks following the injection of Botulinum toxin A into your abdomen, did you notice any changes in the shape of your abdomen?

- The shape of my abdomen did not change.
- I noticed a slight change in the shape of my abdomen.
- I noticed a significant change in the shape of my abdomen.

**4. Physical Function**

**4.1** In the days and weeks after the injection of Botulinum toxin A and up until your hernia surgery, did you notice any differences in your ability to get in and out of bed or to rise from a seated position?

- It was easier.
- I didn't notice any difference.
- It was somewhat more difficult.
- It was much more difficult.

**5. Bowel Movements**

**5.1** In the days and weeks following the injection of Botulinum toxin A and up until the surgery, did you notice any differences in your ability to have a bowel movement?

- It was easier.
- I didn't notice any difference.
- It was somewhat more difficult.
- It was much more difficult.

**6. Urination**

**6.1** In the days and weeks following the Botulinum injection and up until the surgery, did you notice any differences in your ability to urinate?

- It was easier.
- I didn't notice any difference.
- It was somewhat more difficult.
- It was much more difficult.

**7. Open Feedback**

**7.1** Would you like to comment on your experience with the Botulinum injection?
